# Tremor suppression in ECG

**DOI:** 10.1186/1475-925X-7-29

**Published:** 2008-11-19

**Authors:** Ivan A Dotsinsky, Georgy S Mihov

**Affiliations:** 1 Center of Biomedical Engineering, Bulgarian Academy of Sciences, Acad. G. Bonchev str., bl. 105, 1113 Sofia, Bulgaria; 2 Technical University of Sofia, Faculty of Electronic Engineering and Technologies, Kliment Ohridski str. 8, 1000 Sofia, Bulgaria

## Abstract

**Background:**

Electrocardiogram recordings are very often contaminated by high-frequency noise usually power-line interference and EMG disturbances (tremor). Specific method for interference cancellation without affecting the proper ECG components, called subtraction procedure, was developed some two decades ago. Filtering out the tremor remains *a priori *partially successful since it has a relatively wide spectrum, which overlaps the useful ECG frequency band.

**Method:**

The proposed method for tremor suppression implements the following three procedures. Contaminated ECG signals are subjected to moving averaging (comb filter with linear phase characteristic) with first zero set at 50 Hz to suppress tremor and PL interference simultaneously. The reduced peaks of QRS complexes and other relatively high and steep ECG waves are then restored by an introduced by us procedure called linearly-angular, so that the useful high frequency components are preserved in the range specified by the embedded in the ECG instrument filter, usually up to 125 Hz. Finally, a Savitzky-Golay smoothing filter is applied for supplementary tremor suppression outside the QRS complexes.

**Results:**

The results obtained show a low level of the residual EMG disturbances together with negligible distortion of the wave shapes regardless of rhythm and morphology changes.

## Background

Electrocardiogram (ECG) recordings are very often contaminated by residual power-line (PL) interference [1-4], base-line drift [5-7], artefacts and EMG disturbances due to involuntary muscle contractions (tremor) of the patient [8-12]. The base-line drift resulting from electrochemical processes at the electrode-to-skin barrier [7] is a typical low-frequency noise that distorts the susceptible ST segment [6,13]. Interference and tremor have overlapping frequency bands. Therefore, many algorithms are aimed at their common suppression [14-17] in order to provide an accurate automatic delineation of the ECG wave boundaries [18].

Specific digital filter for PL interference cancellation, called subtraction procedure, has been developed some two decades ago and permanently improved later on [19]. It does not affect the signal frequency components around the rated PL frequency. Moving averaging is applied on linear segments of the signal (usually found in the PQ and TP intervals, but also in sufficiently long straight parts of the R and T waves) to remove the interference components. They are stored as phase locked corrections and further subtracted from the signal wherever non-linear segments are encountered, e.g. QRS complexes or other high and steep waves. Several criteria for linearity have been tested and implemented depending on the purpose. In general, they are based on the second difference of the signal (mathematical evaluation of the curvature).

Filtering out the tremor is *a priori *partially successful since it has a relatively wide spectrum, which covers the useful ECG frequency band. One of the first recommendations for ECG instruments [20] suggests a low-pass filter with minimum 35 Hz cut-off. However, in this way the amplitudes of sharp QRS waves are reduced. The moving averaging (comb filter with linear phase characteristic) gives similar results [21].

The time averaging is one of the classic methods for ECG noise suppression. It is based on the assumption that the ECG signal is repeatable [22]. As the variability of the ECG morphology is also suppressed, some authors [23,24] proposed adaptive triggered filtering. Another way to preserve the ECG individuality is to reduce the number of the averaged beats but thus the effect of noise suppression is decreased. The variable ECG morphology, which is related to the respiration, may be compensated in multilead recordings by spatial transformations [24]. However, they can not be applied in the case of single channel time alignment.

Kotas [25] published projective filtering of time-aligned ECG beats. This is an extension of time averaging, which preserves the variability of the beat morphology. The method employs the rules of principal component analysis for the desired ECG reconstruction and aims to retain to some extent the deviations from the averaged component changes, in the same time, rejecting deviations caused by noise. However, the nonlinear projective filtering is computationally intensive and is known to be sensitive to noise changes.

Adaptive filtration has been also attempted but with limited success because the QRS complexes disturb the adaptation process up to the end of the T-waves [14]. Luo and Tompkins [8] obtained faster convergence using additional EMG channel as reference input. Bensadoun *et al *[9] proposed a multidimensional method but the reduction of sharp Q-waves amplitudes is too high.

Clifford *et al *[26] reported a model-based filtering method. P-, Q-, R-, S- and T-waves are defined by a Gaussian with three parameters: amplitude, width and relative position with respect to the R-peak. T-wave is described by T^+ ^and T^- ^because of its asymmetric turning point. Non-linear least-squares optimization is applied to fit this ECG model to the observed signal. The authors present one cleanly recorded P-QRS-T interval superimposed by electrode motion noise. The result shows almost total noise suppression but also significant waveform distortions. However, the locations of the wave peaks match the uncorrupted signal; the errors around the isoelectric line and the S-T segment are negligible. Thus, much of the clinical information of the beats is captured after the noise removal. Nevertheless, the error tolerance has to be tested over a set of databases, since non-parameterized beat will be considered to be an artefact, while some artefacts may closely resemble a known beat. An important advantage of the method is the almost total elimination of series of pulses (artefacts).

Sameni *et al *[27] proposed a nonlinear Bayesian filtering framework consisting of Extended Kalman Filter (EKF), Extended Kalman Smoother (EKS) and Unscented Kalman Filter (UKF) as suboptimal filtering schemes. They are based on modified dynamic ECG model thus utilizing *a priori *information about the underlying dynamics of ECG signals. Recordings taken from the MIT-BIH Normal Sinus Rhythm Database are superimposed by artificially generated noise. They are used for off-line testing EKF, EKS and UKF together with Wavelet denoising technique, adaptive and FIR filtering. A best SNR improvement (difference between output and input SNR) of about 10 dB is obtained with the framework filters. The authors found that brady- or tachycardia do not considerably affect the filter performance, while other abnormalities appearing in some of the ECG cycles may lead to large errors in the Gaussian functions locations. Besides, neither the model nor the measurement is reliable for filtering signals with low input SNR. Therefore, an accurate denoising of abnormal ECGs with high morphological changes remains an open problem.

Christov and Daskalov [10] applied an adopted by Savitzky and Golay [28] smoothing procedure, which uses least square approximation and a special 'wings' function for defining the weighting coefficients. The obtained suppression ration of the EMG artefact is about 6. Low reduction of R and S waves is reported depending of the wave shape.

Nikolaev and Gotchev [11] denoised ECG signals by applying wavelet domain Wiener filtering. They mixed original signals with EMG noise with a SNR = 14 dB. Two-stage algorithm improves the traditional technique by involving time-frequency dependent threshold for calculating the first stage pilot estimate. A SNR over 20 dB is obtained together with less than 10% QRS amplitudes reduction. In another paper Nikolaev *et al *[12] reported an SNR improvement of more than 10 dB.

Another technique for applying the subtraction procedure in the case of tremor is reported by Christov [16]. The approach introduces adaptive criterion for linearity detection based on the ratio *R *between the linear segments length in a selected epoch and its total length usually chosen about 1 s. Normally, the criterion threshold *M *is a constant, which is set from 100 to 160 μV [19]. In the referred publication [16], *M *starts from a low value of 50 μV and increases until *R *reaches a pre-selected value, e.g. 0.9 that corresponds to QRS complex and free of noise RR interval with normal dimensions. The results obtained show a reasonable compromise between tremor suppression and QRS amplitudes reduction.

Gotchev *et al *[29] applied Savitzky-Golay filter inside the QRS complexes and wavelet shrinkage outside them. The first technique gives a good preservation of the RS amplitude of about 30 μV but with low tremor suppression, while the second one offers good suppression with 440 μV decreasing in the RS amplitude. The combined method incorporates the features of both approaches. They are switched depending on the value W of the 'wings' function. W < 10 is taken as dynamic order of the Savitzky-Golay filter; a higher value calls the wavelet subroutine.

When the comb filter is used as a step of the subtraction procedure [19], the signal inside the QRS complexes is not subjected to moving averaging. Thus, the QRS peaks are preserved but in the presence of tremor the complexes become corrupted and the linear segments are not detected correctly, the last leading to: i) unsuppressed disturbance in false non-linear segments, and ii) rare re-calculation of the phase corrections, which can not follow the changes of the interference amplitudes. These problems are overcome to some extent by Dotsinsky and Christov [17], who introduced a parallel buffer. The comb filtering is applied there over the entire signal, thus allowing precise location of the linear segments. However, the possibility of denoising the QRS complexes by inappropriate tremor components as a part of the calculated phase corrections still remains.

## Aim of the study

The purpose of this work was to develop real-time going method and algorithm for suppressing both tremor and PL interference in single- or multilead ECG regardless of SNR, wave shapes and morphology changes.

## Methods and materials

The developed method for tremor suppression in ECG implements the following three procedures:

• Contaminated ECG signals are subjected to moving averaging (comb filter with linear phase characteristic) with first zero set at 50 Hz to suppress tremor and PL interference together.

• The reduced peaks of the processed signal are then restored by an introduced by us procedure called linearly-angular, thus the useful high frequency components are preserved in the range specified by the embedded in the ECG instrument filter, usually up to 125 Hz.

• Finally, a Savitzky-Golay smoothing filter is applied for supplementary tremor suppression outside the QRS complexes.

About 80 episodes consisting of several RR intervals are extracted from 51 AHA database recordings [30]. They are preliminary moving averaged to suppress any undefined inherent noise. The obtained signals are called 'conditionally clean'. The sampling rate is 250 Hz, the resolution is 5 μV/bit.

In the first part of the study the conditionally clean signals are used for developing the recovery procedure and evaluation of its correctness. For this purpose clean signals are comb filtered and then restored. Input and output signals are compared to assess the distortions introduced by the recovery.

In the second part of the study the clean signals are mixed with synthesized 50 Hz PL interference and tremor obtained by two ECG electrodes placed on one forearm. The mixed signals are subjected to all procedures. The obtained results are analysed to evaluate the tremor suppression and PL interference cancellation.

In the third part of the study the procedures are applied directly on noisy recordings taken from the AHA database and MIT-BIH Noise Stress Database.

## Signal recovery

### Basic relations between filtered and non-filtered samples

The formulae for calculating the middle term in moving averaging over *n *samples for odd *n *= 2*m*+1 and even *n *= 2*m *[19] are presented below:

(1) $$\begin{array}{cccc} Y_{i}=\frac{1}{n}\sum_{j=-m}^{m}X_{i+j}, & n=2m+1; & Y_{i}=\frac{1}{n}\left[\sum_{j=-\left(m-1\right)}^{m-1}X_{i+j}+\frac{X_{i+m}+X_{i-m}}{2}\right], & n=2m \end{array}.$$

Here *m *is integer, *n *is equal to the sampling rate divided by the rated interference frequency; *i *stands for the position of the ongoing averaged sample *Y*_*i*_, which is obtained over **m **surrounding non-averaged samples.

Taking in consideration that $\sum_{j=-m}^{m}X_{i+j}=\sum_{j=-m}^{-1}X_{i+j}+X_{i}+\sum_{j=1}^{m}X_{i+j}$ and $\sum_{j=-m}^{-1}X_{i+j}=\sum_{j=1}^{m}X_{i-j}$, equation (1) can be expressed by

(2) $$\begin{array}{ll} Y_{i}=\frac{1}{n}\left[\sum_{j=1}^{m}\left(X_{i-j}+X_{i+j}\right)+X_{i}\right], & n=2m+1\\ Y_{i}=\frac{1}{n}\left[\sum_{j=1}^{m-1}\left(X_{i-j}+X_{i+j}\right)+\frac{X_{i-m}+X_{i+m}}{2}+X_{i}\right], & n=2m \end{array}.$$

Substituting $X_{i}=nX_{i}-2\sum_{j=1}^{m}X_{i}$, *n *= 2*m *+ 1; $X_{i}=nX_{i}-2\left(\sum_{j=1}^{m-1}X_{i}+\frac{X_{i}}{2}\right)$, *n *= 2*m*, equation (2) is transformed in

(3) $$\begin{array}{ll} Y_{i}=X_{i}+\frac{1}{n}\left[\sum_{j=1}^{m}\left(X_{i+j}-2X_{i}+X_{i-j}\right)\right], & n=2m+1\\ Y_{i}=X_{i}+\frac{1}{n}\left[\sum_{j=1}^{m-1}\left(X_{i+j}-2X_{i}+X_{i-j}\right)+\frac{X_{i+m}-2X_{i}+X_{i-m}}{2}\right], & n=2m \end{array}.$$

The polynomial inside the parentheses is a second difference, represents one of the possible versions of the linear criterion [19] and is further denoted as

*D*_*i*,*j *_= *X*_*i*+*j *_- 2*X*_*i *_+ *X*_*i*-*j *_= (*X*_*i*+*j *_- *X*_*i*_) - (*X*_*i *_- *X*_*i*-*j*_). Using this equation, the samples *X*_*i *_and *Y*_*i *_can be expressed by

(4) $$\begin{array}{cccc} Y_{i}=X_{i}+\frac{1}{n}\sum_{j=1}^{m}D_{i,j}, & n=2m+1; & Y_{i}=X_{i}+\frac{1}{n}\left(\sum_{j=1}^{m-1}D_{i,j}+\frac{D_{i,m}}{2}\right), & n=2m \end{array}.$$

The mean signal velocities on the left and the right hand side of the ongoing sample *X*_*i *_are $v_{i,i-j}=\frac{X_{i}-X_{i-j}}{j}$ and $v_{i+j,i}=\frac{X_{i+j}-X_{i}}{j}$. They are averaged within the intervals [*i-j*, *i*] and [*i*, *i+j*], since they correspond to the time-coordinates *i+j*/2 and *i-j*/2.

Then, equation (4) is presented as

(5) $$\begin{array}{ll} Y_{i}=X_{i}+\frac{1}{n}\sum_{j=1}^{m}j\left(v_{i+j,i}-v_{i,i-j}\right), & n=2m+1\\ Y_{i}=X_{i}+\frac{1}{n}\left[\sum_{j=1}^{m-1}j\left(v_{i+j,i}-v_{i,i-j}\right)+\frac{m\left(v_{i+m,i}-v_{i,i-m}\right)}{2}\right], & n=2m \end{array}.$$

### Background of the linearly-angular recovery procedure

**Figure 1:**
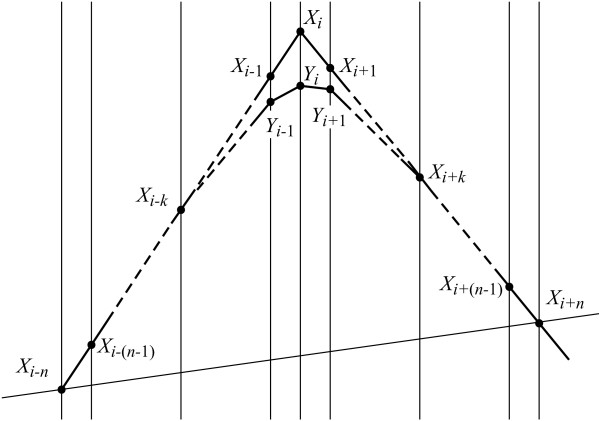
Linearly-angular recovery of the signal in the interval [i-*n*, ..., i+*n*].

Let us assume that the conditionally clean signal is linear aside from the ongoing sample *X*_*i *_and has a triangular-like shape (Fig. 1). Then *v*_*i*+*j*,*i *_= *v*_*r*_, *j *= *i*+1, ..., *i*+*n*; *v*_*i*+*j*,*i *_= *v*_*l*_, *j *= *i*-*n*, ..., *i*-1 and the difference *v*_*i*+*j*,*i *_- *v*_*i*,*i*-*j *_= *v*_*r *_- *v*_*l *_as well as the ratio *D*_*i*,*j*_/*j *= *D*_*i*,*k*_/*k*, *k *= 1, 2, ..., *n *are constant.

The equation (5) is transformed into a uniform expression both for odd and even number of averaged samples:

(6) $$Y_{i}=X_{i}+(v_{r}-v_{l})\kappa_{n}=X_{i}+\frac{D_{i,k}}{k}(v_{r}-v_{l})\kappa_{n},$$

where the constant κ_*n *_is given by

(7) $$\begin{array}{cccc} \kappa_{n}=\frac{1}{n}\sum_{j=1}^{m}j=\frac{n^{2}-1}{8n}, & n=2m+1; & \kappa_{n}=\frac{1}{n}\left(\sum_{j=1}^{m-1}j+m\right)=\frac{n}{8}, & n=2m. \end{array}$$

Equation (6) can be written as

(8) $$X_{i}=Y_{i}-D_{i,k}\frac{\kappa_{n}}{k}=Y_{i}-D_{i,k}K,$$

Analogously to the second difference *D*_*i*,*k*_, a filtered second difference $D_{i,k}^{*}$ = *Y*_*i*+*k *_- 2*Y*_*i *_+ *Y*_*i*-*k *_is introduced using filtered signal samples. Substituting *D*_*i*,*k *_= η$D_{i,k}^{*}$, the back filtered sample X*_i _can be calculated by

(9) $$X_{i}^{*}=Y_{i}-\eta D_{i,k}^{*}K,$$

The coefficient η is intended to consider the real signal shapes. For the time being, this study presumes that η is very close to 1.

The influence of *k *on the back filtering error is assessed by experiments with *k *= 1, 2, 3, 4, 5; *n *= 5 and *M *= 0,12 *mV*. The error committed is minimal with *k *= 2, which value is further used. Lower value of *k *contributes to better shape recovery of rounded peaks, while the steeper ones are sub-compensated. Higher *k *value restores well steep peaks, but the rounded ones become over-compensated.

### Assessment of the recovery procedure

**Figure 2:**
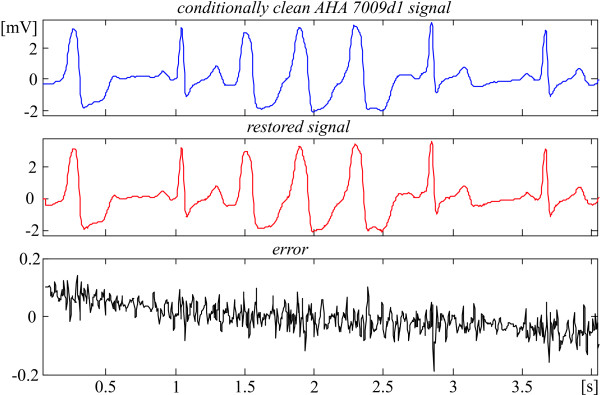
Comparison between 'clean' and restored AHA 7009d1 episode.

**Figure 3:**
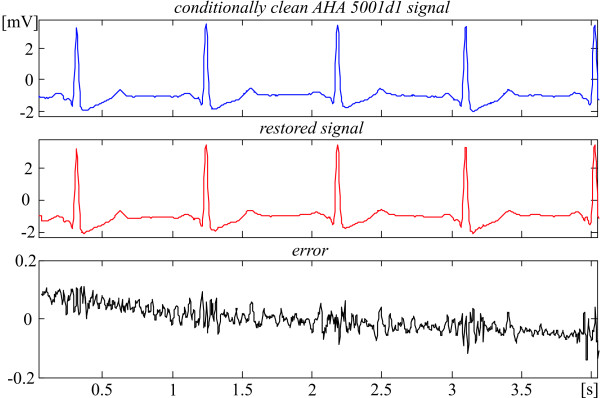
Comparison between 'clean' and restored AHA 5001d1 episode.

**Figure 4:**
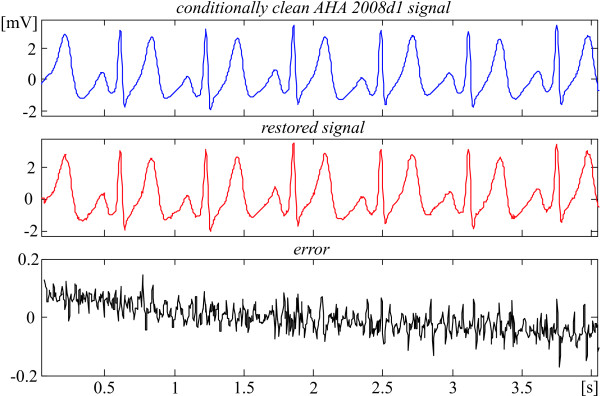
Comparison between 'clean' and restored AHA 2008d1 episode.

**Figure 5:**
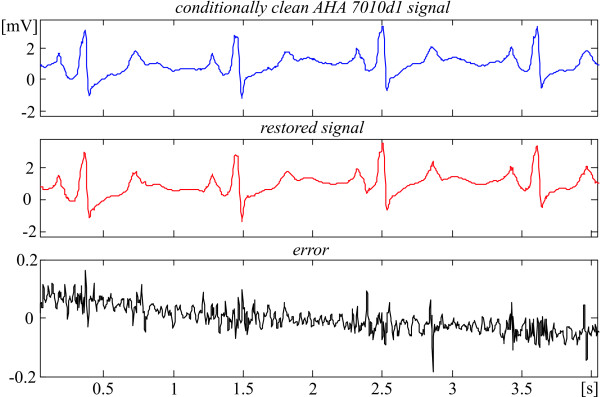
Comparison between 'clean' and restored AHA 7010d1 episode.

The recovery evaluation is illustrated by episodes of some AHA signals shown in Fig. 2, 3, 4, 5. They present different ECG rhythm and wave shapes: QRS complexes + ectopic beats (Fig. 2), high and steep QRS complexes (Fig. 3), high T waves (Fig. 4), high P wave + ST depression (Fig. 5). The two upper traces are clean and processed signals, respectively. The lower traces demonstrate an error committed in the range of 3%. No loss of clinical information is observed. The results obtained with the other episodes taken from the 51 AHA recordings are identical or better. These episodes are listed in Table 1 with their starting and ending times.

**Table 1:** Starting and ending times of the AHA recordings used for assessment of the recovery procedure.

	episode taken			episode taken			episode taken	
					
AHA recording	starting time, s	ending time, s	AHA recording	starting time, s	ending time, s	AHA recording	starting time, s	ending time, s
1004d1	384	416	4001d2	1152	1176	6008d1	864	896

1010d1	672	704	4005d1	192	224	6009d1	864	952

1010d2	672	704	4005d2	192	288	6009d1	1056	1112

2001d1	672	688	4006d1	384	400	6010d1	1056	1088

2004d1	1056	1072	4006d1	768	784	6010d1	1152	1176

2004d1	1152	1168	4006d2	384	400	7001d2	480	552

2004d1	1248	11296	4006d2	768	784	7002d1	280	512

2004d2	1056	1072	4009d1	288	336	7002d1	576	608

2004d2	1152	1168	4009d2	288	336	7003d2	768	800

2004d2	1248	11296	5001d1	9	65	7004d1	288	320

2005d1	1	41	5001d1	768	824	7005d1	480	560

2005d2	1	41	5003d1	864	920	7005d1	576	656

2008d1	9	57	5003d1	960	1016	7005d2	480	512

2008d1	864	888	5003d1	1056	1104	7005d2	576	608

2008d1	1056	1080	5003d2	960	1040	7006d1	672	728

2008d2	9	57	5004d2	288	312	7006d2	672	696

2008d2	864	888	5009d2	576	624	7007d1	672	760

2008d2	1056	1080	5010d1	864	896	7007d2	672	720

2009d1	1152	1208	5010d1	960	992	7008d1	288	320

2009d2	1152	1208	5010d1	1152	1184	7009d1	9	65

3004d1	576	632	5010d2	960	992	7009d1	96	128

3004d2	576	632	6002d1	288	328	7009d1	960	992

4001d1	960	1016	6002d2	288	304	7010d1	672	752

4001d1	1056	1088	6003d2	288	344	7010d1	768	824

4001d1	1152	1176	6005d1	192	280	7010d1	864	896

4001d2	960	1016	6007d1	1056	1080	7010d2	864	896

4001d2	1056	1088	6007d2	1056	1080	7010d2	1152	1184

The recovery is assessed without additional suppression outside the QRS complexes in order to have statistically the same residual noise all over the episode. Thus, a more accurate evaluation of the distortions within the complexes is possible.

Actually, the linear segments outside the ventricular beats (see for example Fig. 2 and 3) that represent physiological zero-line should be free of any distortions. Obviously, the 'error' there is due to noise components of the AHA recordings that have not been totally eliminated by the preliminary moving averaging, since the first lobe of the comb filter [21] has an equivalent high-pass cut-off approximately at 24 Hz.

This impression may be reinforced by visual inspection of tremor episodes after moving averaging followed by some kind of additional filtering.

Consequently, the real errors own to the procedure are considerably smaller. One may speculate that the distortions introduced by the recovery inside the QRS complexes are within ± 50 μV (see Fig. 2, 3, 4, 5).

## Additional tremor suppression in the linear segments

**Figure 6:**
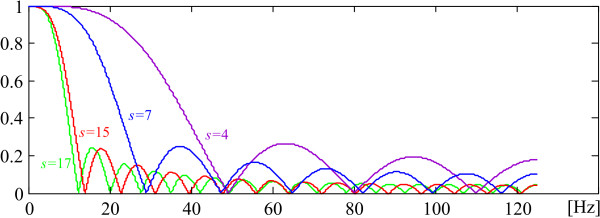
Savitzky-Golay frequency responses obtained with different parameter *s*.

Fig. 6 shows Savitzky-Golay frequency responses obtained for 250 Hz sampling rate with different parameter *s*. Here the original notation *n *[28] is substituted by *s *in order to avoid confusion with the number of samples in one PL period.

$$\begin{array}{ccc} Y_{i}=\frac{1}{N}\sum_{j=-s}^{s}C_{j}X_{i+j}, & C_{j}=3s^{2}+3s-1-5j^{2}, & N=\left(2s+1\right)\left(4s^{2}+4s-3\right)/3 \end{array}$$

Filters with parameter *s *< 4 are unusable since their first zero is shifted too far towards the high frequencies that stultify the attempts for tremor suppression. In this study s = 15 is used as a compromise between good tremor suppression and preserving the P-wave shapes.

**Figure 7:**
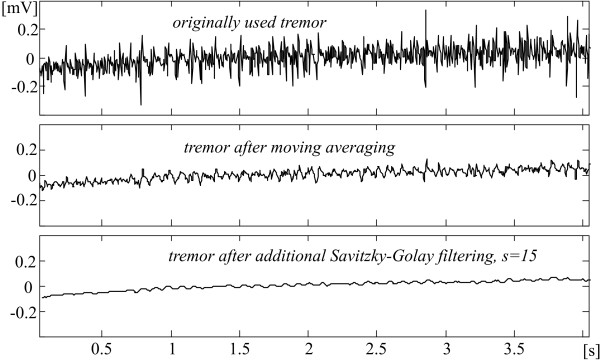
Reduction of the tremor amplitudes after moving averaging and Savitzky-Golay filter.

**Figure 8:**
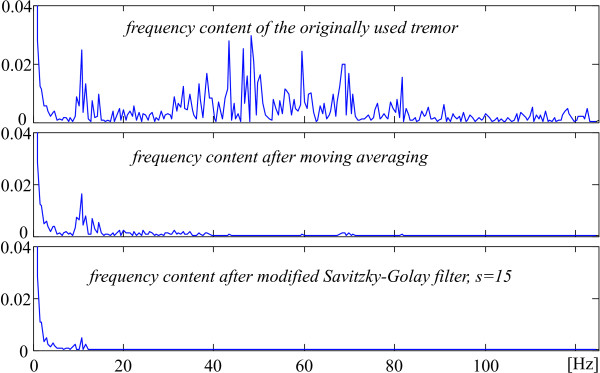
Frequency content of the tremor after moving averaging and Savitzky-Golay filter.

The expected effect of the additional tremor suppression outside the QRS complexes and some high T-waves can be seen in Fig. 7 and 8. The first one shows a considerable tremor amplitude reduction after the moving averaging and the Savitzky-Golay filter. Therefore, a part of the residual tremor in the processed signals demonstrated below, which are taken from the AHA database, is due to noise components in the original recordings. Fig. 8 presents the FFT diagrams of the two consecutive filtrations.

The observation of the traces in Fig. 7 suggests how to assess the suppression ratio of both procedures. It is quite possible that the maximum peak coupled to a relatively high frequency before filtering is well suppressed after filtering while a lower amplitude lower frequency peak before may practically preserve its amplitude after that. Therefore, the suppression ratio could be defined as the quotient of the maximum peaks in signals before and after processing. For the moving averaging such ratio is over 6 times. It becomes about 25 after additional Savitzky-Golay filtering.

## Results

### Evaluation of the noise suppression in conditionally clean signals mixed with PL interference and tremor

**Figure 9:**
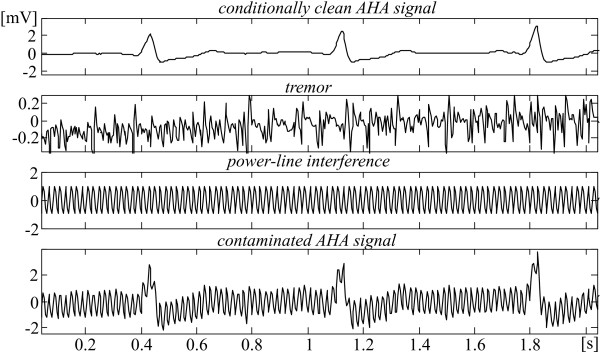
Contaminated episode ('clean' signal + tremor+ interference).

Fig. 9 illustrates how the contaminated signals are obtained. A conditionally clean ECG episode (upper trace) is mixed with tremor (second trace) and interference (third trace) to be used further (lower trace) for precise assessing the tremor suppression and PL interference cancellation when the three procedures are applied.

**Figure 10:**
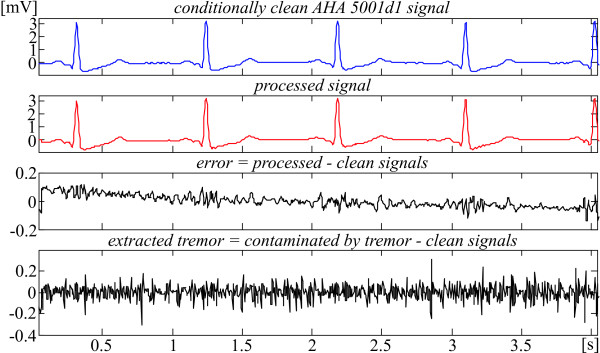
Differences between contaminated and processed AHA 5001d1 signal.

Noise suppression of the contaminated AHA 5001d1 episode is presented in Fig. 10. It is chosen for comparison with Fig. 3, where the same clean signal is used as input. The traces are as follows: conditionally clean signal; processed signal; error = processed - clean signals; extracted tremor = contaminated by tremor - clean signals. The PL interference is totally eliminated [19]. For more clarity, the contaminated signal is not shown. The error within the QRS complexes is the same as presented in Fig. 3. The tremor suppression outside the complexes is higher due to the additional Savitzky-Golay filtering. The extracted tremor is a considerable part of the non-correlated added and residual tremors of the clean signal. Again, all clinical information (P waves, QRS parameters, ST segments, T waves) is preserved. This is true also for the other contaminated and tested AHA database episodes.

**Figure 11:**
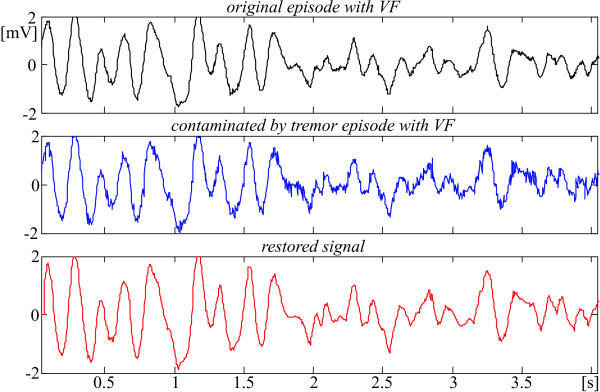
Tremor suppression in episode with VF taken from AHA 8003d1 recording, starting at 1161 s.

**Figure 12:**
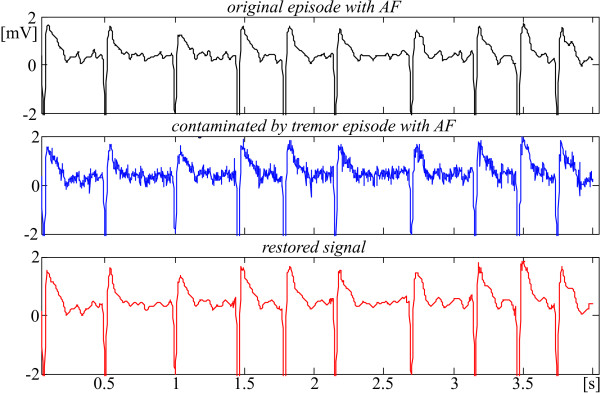
Tremor suppression in episode with AF taken from MIT-BIH Atrial Fibrillation/Flutter database, 04936 recording, starting at 27 min and 18 s.

The next Fig. 11 and 12 demonstrate how the identification marks of some specific rhythms such atrial and ventricular fibrillation are preserved (see for example the f-wave shapes in Fig. 12).

### Tremor suppression in originally noisy recordings

**Figure 13:**
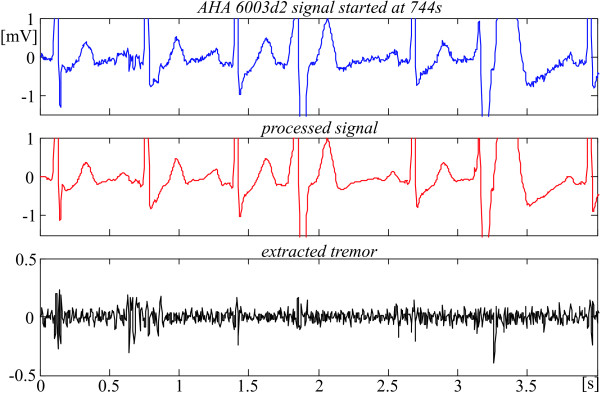
Noisy suppression in AHA 6003d2 episode.

**Figure 14:**
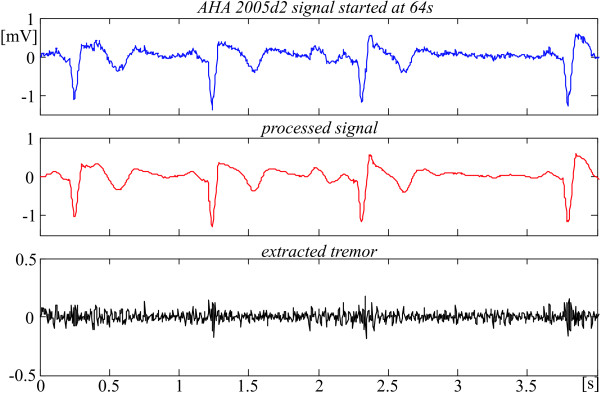
Noisy suppression in AHA 2005d2 episode.

The efficiency of the reported method and algorithm is illustrated below by two originally noisy AHA recordings subjected to the procedures (figures 13 and 14). The two first traces are the original and the processed signals, respectively. The lower traces point out the extracted tremor.

**Figure 15:**
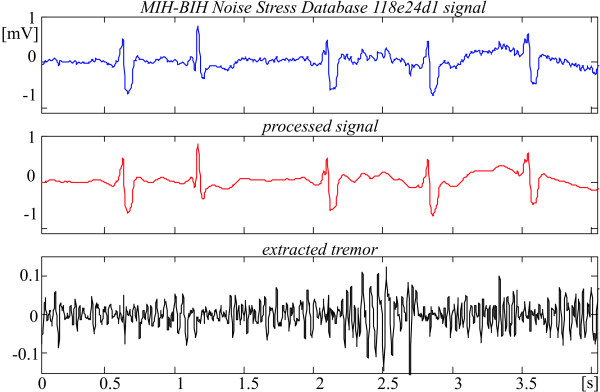
Noisy suppression of 118e24d1 Noise Stress Database episode, starting at 499 s.

**Figure 16:**
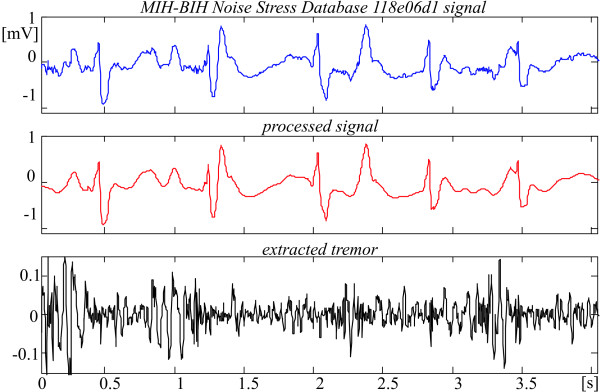
Noisy suppression of 118e06d1 Noise Stress Database episode, starting at 306 s.

Fig. 15 and 16 show the result of tremor suppression in episodes taken from the MIT-BIH Noise Stress Database.

## Discussion and conclusion

The proposed method for tremor suppression in one- or multilead ECG is based on moving averaging of the ECG signal followed by a linearly-angular procedure for restoring the affected amplitudes of QRS complexes and other relatively high and steep ECG waves. Thus, the useful high frequency components are preserved in the range specified by the embedded in the ECG instrument filter, usually up to 125 Hz. Finally, the signal portions outside the QRS complexes are additionally processed to reduce the tremor level by applying a Savitzky-Golay smoothing procedure. The results prove the efficiency of the developed method. The recovery error of about 50 μV is below the level that may provoke wrong diagnostic. The interference is totally eliminated. The tremor is suppressed approximately 25 times. The residual tremor does not lead to false ECG interpretation. The procedure efficiency is independent on arrhythmia and any other wave shape variations. The algorithm is suitable for real-time implementation. For the time being the individual shape of the restored waves (the coefficient η) is not taken in consideration. This possibility will be further checked up.

## Competing interests

The authors declare that they have no competing interests.

## Authors' contributions

GM developed the recovery procedure and determined the optimal order of the Savitzky-Golay filter. ID carried out the other parts of the study. Both authors discussed the ideas accompanying the elaboration of the reported method for tremor suppression in ECG. They read and approved the final manuscript.

## References

[B1] Huhta JC, Webster JG (1973). 60 Hz interference in electrocardiography. IEEE Trans Biomed Eng.

[B2] van Metting R, Peper A, Grimbergen CA (1990). High-quality recording of bioelectrical events, Part 1: Interference reduction, theory and practice. Med Biol Eng Comput.

[B3] Ziarani AK, Konrad A (2002). A Nonlinear Adaptive Method of Elimination of Power Line Interference in ECG Signals. IEEE Trans Biomed Eng.

[B4] Ju-Won Lee, Gun-Ki Lee (2005). Design of an adaptive filter with a dynamic structure for ECG signal processing. Intern J Contr Automat Syst.

[B5] Pottala EW, Bailey JJ, Horton MR, Gradwohl JR (1989). Suppression of baseline wander in the ECG using a bilinearly transformed, null phase filter. J Electrocardiol.

[B6] Frankel RA, Pottala EW, Browser RW, Bailey JJ (1991). A filter to suppress ECG baseline wander and preserve ST-segment accuracy in a real-time environment. J Electrocardiol.

[B7] McAdams ET, Jossinet J (2000). Nonlinear transient response of electrode-electrolyte interface. Med Biol Eng Comput.

[B8] Luo S, Tompkins WJ (1995). Experimental study: Brachial motion artefact reduction in the ECG. Comp Cardiol.

[B9] Bensadoun Y, Novakov E, Raoof K, Roberge FA, Kearney RE (1995). Multidimensional adaptive method for cancellation EMG signals from the ECG signal. Proceedings of the 17th Annual International Conference on the IEEE Engng in Med and Biol Soc 1995: Montreal.

[B10] Christov II, Daskalov IK (1999). Filtering of electromyogram artifacts from the electrocardiogram. Med Eng Phys.

[B11] Nikolaev N, Gotchev A (2000). ECG signal denoising using wavelet domain Wiener filtering. Proceedings of the European Signal Processing Conference EUSIPCO-2000.

[B12] Nikolaev N, Gotchev A, Egiazarian K, Nikolov Z (2001). Suppression of electromyogram interference on the electrocardiogram by transform domain denoising. Med Biol Eng Comput.

[B13] Dotsinsky I, Stoyanov T (2004). Optimization of bi-directional digital filtering for drift suppression in electrocardiogram signals. J Med Eng Technol.

[B14] Thakor NV, Zhu Y (1991). Applications of adaptive filtering to ECG analysis: noise cancellation and arrhythmia detection. IEEE Trans Biomed Eng.

[B15] Romanca M, Szabo W (1998). Electrocardiogram pre-processing for the removal of high frequency and power-line frequency noise. Proceedings of the 6th International Conference on Optimization of Electrical and Electronic Equipments: May 1998; Braşov.

[B16] Christov II (2000). Dynamic power-line interference subtraction from biosignals. J Med Eng Techn.

[B17] Dotsinsky I, Christov I (2002). Power-line interference subtraction from the electrocardiogram in the presence of electromyogram artifacts. Electrotechnika Elektronika.

[B18] Sahambi JS, Tandon SN, Bhatt RKP (1997). Quantitative analysis of errors due to power line interference and base line drift in detection of onsets and offset in ECG using wavelets. Med Biol Eng Comput.

[B19] Levkov C, Mihov G, Ivanov R, Daskalov I, Christov I, Dotsinsky I (2005). Removal of Power-line Interference from the ECG: a Review of the Subtraction Procedure. BioMed Eng OnLine.

[B20] (1967). Subcommittee on Instrumentation Committee on Electrocardiography – American Heart Association, Recommendation for instruments in electrocardiography and vectorcardiography. IEEE Trans Biomed Eng.

[B21] Lynn PA (1977). Online digital filters for biological signals: some fast designs for a small computer. Med Biol Eng Comput.

[B22] Jane R, Rix H, Caminal R, Laguna P (1991). Alignment methods for averaging of high resolution cardiac signals: A comparative study of performance. IEEE Trans Biomed Eng.

[B23] Laguna P, Jane R, Meste O, Poon PW, Caminal P, Rix H, Thakor NV (1992). Adaptive filter for event-related bioelectric signals using impulse correlated reference input: Comparison with signal averaging techniques. IEEE Trans Biomed Eng.

[B24] Aström M, Carro Santos E, Sörnmo L, Laguna P, Wohlfar B (2000). Vectorcardiographic loop alignment and the measurement of morphologic beat-to-beat variability in noisy signals. IEEE Trans Biomed Eng.

[B25] Kotas M (2004). Projective filtering of time-aligned ECG beats. IEEE Trans Biomed Eng.

[B26] Clifford GD, Shoeb A, McSharry PE, Janz BA (2005). Model-based filtering, compression and classification of the ECG. Intern J Bioelectromagnetism.

[B27] Sameni R (2007). A nonlinear Bayesian filtering framework for ECG denoising. IEEE Trans Biomed Eng.

[B28] Savitzky A, Golay M (1964). Smoothing and differentiation of data by simplified least square procedures. Anal Chem.

[B29] Gotchev A, Christov I, Egiazarian K (2002). Denoising the electrocardiogram from electromyogram artifacts by combined transform-domain and dynamic approximation method. proceedings of the International Conference on Acoustics Speech and Signal Processing ICASSP'2002: Orlando USA 13–17 May.

[B30] American Heart Association (AHA) arrhythmia ECG database. Emergency care Research Institute 5200 Butler Pike, Plymouth Meeting, PA 19462 USA.

